# Editorial for the Special Issue “Acute and Chronic Pancreatitis, Pancreatic Malignancies”

**DOI:** 10.3390/medicina59050984

**Published:** 2023-05-19

**Authors:** Antanas Gulbinas, Povilas Ignatavicius, Zilvinas Dambrauskas

**Affiliations:** 1 Department of Surgery, Medical Academy, Lithuanian University of Health Sciences, 44307 Kaunas, Lithuania; antanas.gulbinas@lsmuni.lt (A.G.); povilas.ignatavicius@lsmuni.lt (P.I.); 2 Institute for Digestive Research, Medical Academy, Lithuanian University of Health Sciences, 44307 Kaunas, Lithuania

Pancreatic diseases, especially acute pancreatitis and pancreatic cancer, are associated with high rates of complications, difficult treatment that may not always be effective, and high mortality in complex cases. Therefore, this Special Issue aims to discuss the current trends in the diagnosis and management of pancreatic diseases, and share the most recent findings from genetic, molecular, and clinical studies in pancreatology, including acute pancreatitis, cystic lesions, and pancreatic cancer. We are proud to introduce several papers presenting cutting-edge research focusing on the molecular mechanisms of pancreatic diseases, and novel approaches to the diagnosis, prognostication, and treatment of acute pancreatitis and pancreatic malignancies.

Acute pancreatitis (AP) is an insidious, potentially fatal disease affecting not only the pancreas and surrounding tissues, but also other internal organs and organ systems. While most patients with AP have a mild course and the disease is self-limiting, about 20% of AP patients progress to severe disease [1]. When choosing treatment options for acute pancreatitis, several challenges and disagreements arise. If severe acute pancreatitis (SAP) with organ failure develops, patients often need to be transferred to the intensive care unit. Therefore, it is essential to recognize severe disease in the early phase of AP and select patients who would benefit from early interventions [1].

Pancreatic acinar cell injury triggers the release of pro-inflammatory cytokines and chemokines. Subsequently, this initiates an acute inflammatory response, in a manner that is similar to the molecular/signaling events observed in sepsis. The secretion patterns of pro- and anti-inflammatory cytokines have been analyzed in numerous clinical and experimental studies. Most of them show that the deregulation of the cellular immune system is a key event leading to severe AP [2]. However, the study by Zhou R et al. showed that the trend of cytokine expression in rats with SAP was not consistent with the disease progression, and the dominant cytokine–cytokine receptor interactions were always highly expressed at various time points over the course of SAP [3].

Several methods for estimating the severity of AP are widely used today and include APACHE II, the Imrie and Ranson scores, the CT scoring system, and measurement of C-reactive protein and a number of laboratory markers [4,5,6,7,8,9,10]. The current Special Issue includes a study by Sui Y et al. that confirms the possibility of using Fibrinogen-like Protein 1 as a predictive marker for the stratification of AP and its infectious complications [11]. The Controlling Nutritional Status (CONUT) score and prognostic nutritional index (PNI) were proven to be useful prognostic markers not only for predicting nutritional status but also for estimating the severity and outcomes of AP [12].

Early and accurate prediction of disease severity is one of the first steps when choosing the optimal treatment [13]. Identification of the location and extent of pancreatic necrosis could predict specific complications, such as fluid collections [14]. The study by Dekeryte et al. concluded that in patients with pancreatic necrosis exceeding 50%, the clinical course and outcomes were worse. These patients most often developed severe AP, spent more time in the hospital and ICU, and more often needed surgical interventions with more complex treatment. Therefore, timely diagnosis of pancreatic necrosis and evaluation of its volume and extent are highly important in the management of patients with acute necrotizing pancreatitis [15]. Accurate radiological evaluation is of utmost importance for choosing the treatment options when walled-off pancreatic necrosis (WON) is present. This type of necrosis can be intra-pancreatic, peri-pancreatic, or both. Several studies have reported that the following minimally invasive approaches can achieve better outcomes: endoscopic transluminal drainage (ETD) with or without necrosectomy, laparoscopic or retroperitoneal surgical approach, and radiology-guided percutaneous approach followed by necrosectomy [16,17,18]. However, the study by Pattarapuntakul T et al. highlights the advantages of endoscopic transluminal drainage (ETD) with or without necrosectomy [19].

Intraductal papillary mucinous neoplasm (IPMN) is a potentially premalignant lesion of the pancreas, and patients diagnosed with main-duct and mixed-type IPMN, in particular, are often scheduled for pancreatic resection. Analysis of the 5-year experience of the Department of Digestive Tract Surgery of the Medical University of Silesia, Katowice, Poland, revealed that IPMN was the most frequent resected pancreatic cystic tumor (PCT). In patients with PCTs, due to substantial postoperative morbidity, adequate patient selection, considering both the surgical risk and the long-term risk of malignant transformation, is very important. The analysis showed that the distribution of different types of PCTs in a large Eastern European center was similar to that in North American and Western European populations, but differed from that in Eastern (Indian and Chinese) populations [20].

This Special Issue incorporates three review papers on pancreatic tumors stating that lifestyle seems to be a major contributor to the development of pancreatic cancer. Special attention should be given to individuals with a vicious cluster consisting of metabolic syndrome, tobacco smoking, and alcohol consumption [21]. Moreover, the multidisciplinary approach of establishing pancreatic cancer resectability [22] and prognostication of pancreatic fistulas following resection with regard to early postoperative hypophosphatemia is discussed [23].

Unresectable pancreatic cancer results in extremely low rates of survival; therefore, management of the leading complications such as biliary obstruction should be carried out under a minimally invasive but effective approach. Currently, the standard approach in those cases is endoscopic stenting via endoscopic retrograde cholangiopancreatography (ERCP). EUS-guided choledochoduodenostomy is an alternative option for palliative management of malignant distal biliary obstruction [24].

In summary, this Special Issue presents the latest evidence concerning the mechanisms, diagnosis, and management of pancreatic diseases. The development of novel strategies in the management of pancreatic diseases is increasingly moving towards the personalized medicine direction. This is the only way of future management of these challenging conditions.
